# Morphological characterization of domatium development in *Callicarpa saccata*

**DOI:** 10.1093/aob/mcz193

**Published:** 2019-11-26

**Authors:** Emma Sarath, Kazune Ezaki, Takenori Sasaki, Yu Maekawa, Yuji Sawada, Masami Yokota Hirai, Akiko Soejima, Hirokazu Tsukaya

**Affiliations:** 1 Graduate School of Science, The University of Tokyo, Bunkyo-ku, Tokyo, Japan; 2 The University Museum, The University of Tokyo, Bunkyo-ku, Tokyo, Japan; 3 RIKEN Center for Sustainable Resource Science, Yokohama, Japan; 4 Division of Biological Science, Graduate School of Science and Technology, Kumamoto University, Kumamoto, Japan; 5 ExCELLS, National Institutes of Natural Sciences, Okazaki, Japan

**Keywords:** Ant plant, *Callicarpa saccata*, *Callicarpa subaequalis*, development, domatium, gland, Lamiaceae, morphology, mutualism, myrmecophyte, symbiotic

## Abstract

**Background and aims:**

Domatia are plant structures within which organisms reside. *Callicarpa saccata* (Lamiaceae) is the sole myrmecophyte, or ‘ant plant’, that develops foliar (leaf-borne) myrmeco-domatia in this genus. In this work we examined domatium development in *C. saccata* to understand the developmental processes behind pouch-like domatia.

**Methods:**

Scanning electron microscopy, sectioning and microcomputed tomography were carried out to compare the leaves of *C. saccata* with those of the closely related but domatia-less myrmecophyte *Callicarpa subaequalis*, both under cultivation without ants.

**Key results:**

*Callicarpa saccata* domatia are formed as a result of excess cell proliferation at the blade/petiole junctions of leaf primordia. Blade/petiole junctions are important meristematic sites in simple leaf organogenesis. We also found that the mesophyll tissue of domatia does not clearly differentiate into palisade and spongy layers.

**Conclusions:**

Rather than curling of the leaf margins, a perturbation of the normal functioning of the blade/petiole junction results in the formation of domatium tissue. Excess cell proliferation warps the shape of the blade and disturbs the development of the proximal–distal axis. This process leads to the generation of distinct structures that facilitate interaction between *C. saccata* and ants.

## INTRODUCTION

Domatia are structures that are frequently found in plants in tropical regions and have strong associations with organisms such as ants and mites. Unlike galls, domatia are usually formed by plants alone, although some examples of domatia triggered by insects do exist (Blüthgen and Wesenberg, 2001; Tepe *et al*., 2007). Domatia that facilitate interactions with mites are known as acarodomatia. Although some acarodomatia act antagonistically with their inhabitants (Nishida *et al.*, 2005), many plant–mite associations are mutualistic, with predatory or microbivorous mites gaining protection from environmental stresses and predatory arthropods (Grostal and O’Dowd, 1994; Norton *et al.*, 2001; Agrawal, 2003) in exchange for reducing the number of herbivorous mites or insects and protecting the domatium-bearing plants from damage (Grostal and O’Dowd, 1994; Agrawal *et al.*, 2003).

2 Myrmecophytes are plants that associate with ants; one of the best-known examples is the *Vachellia–Pseudomyrmex* relationship, which was first reported in 1966 (Janzen, 1966). The domatia of these ‘ant-loving’ plants are known as myrmeco-domatia. More than 600 plant species exhibit such interactions with ants (Chomicki and Renner, 2015) in symbiotic relationships. Plants often provide shelter and, in some cases, nutrients in exchange for protection from competitors and phytophagous herbivores (Janzen, 1966; Fiala *et al*., 1989; Fiala and Maschwitz, 1992; Vasconcelos, 1991; Michelangeli, 2006).

3 In some species, domatia may be formed through a combination of plant and insect activity. In *Vochysia vismiaefolia*, ants induce domatia through tissue excavation (Blüthgen and Wesenberg, 2001). In the genus *Piper*, there are anatomical differences between the pith of myrmecophytic and non-myrmecophytic species that allow ants to easily excavate the stems of myrmecophytic plants (Tepe *et al.*, 2007). There are two types of domatia: primary and secondary. Primary domatia are formed by modification of naturally existing plant structures (e.g. hollow stems), whereas secondary domatia, such as the foliar domatia that are the focus of this study, can be considered to be organs in their own right (Benson, 1985).

4 Whereas the majority of myrmeco-domatia are stem-based, numerous examples of independent evolution of foliar domatia have been described (Chomicki and Renner, 2015). The present study focuses on ‘sac-like’ or ‘pouch-like’ foliar myrmeco-domatia. Such domatia are found in plants of the Chrysobalanaceae (*Hirtella*), Lamiaceae (*Callicarpa*), Melastomataceae (*Clidemia*, *Conostegia*, *Maieta*, *Miconia*, *Tococa*), Rubiaceae (*Duroia*, *Ixora*) and Malvaceae (*Cola*), many of which have been described from anatomical or morphological standpoints (Vasconcelos, 1991; Svoma and Morawetz, 1992; Nickol, 1993; Janka *et al.*, 2000; Izzo and Vasconcelos, 2002; Leroy *et al.*, 2008, 2010; Bramley, 2009; Michelangeli, 2010; Vogel, 2012; Cárdenas *et al*., 2014; Kriebel, 2016; Nakashima *et al.*, 2016).

5 Pouch-like domatium morphology differs in several ways among species. The development of myrmeco-domatia in *Hirtella physophora* was formerly reported to be due to the curling under of the blade, resulting in the creation of a cavity on either side of the petiole (Leroy *et al.*, 2008). Domatia of this type are known as rolled-margin domatia or ‘domatia revoluta’ (Stace, 1965). In *H. physophora,* a developmentally intermediate stage of curling is seen prior to the formation of a mature domatium. At this stage, the leaf margins have folded downwards but not yet connected to the midvein, leaving domatia open along the proximal–distal axis (Leroy *et al.*, 2008). The Brazilian pepper-tree species *Schinus terebinthifolia* (Anacardiaceae) possesses acarodomatia that also form through folding (Wiggers *et al.*, 2005; Pireda *et al.*, 2017); partially developed domatia are observed before the final domatium form emerges. On the contrary, in the aforementioned genera *Tococa* and *Maieta* (Melastomataceae), domatia develop without transitional forms (Leroy *et al.*, 2008). Additionally, the domatia of *Maieta guianensis* are present only in the proximal leaf tissue closest to the midvein, while the marginal regions of the blade are flat (Leroy *et al.*, 2010). In *Callicarpa saccata*, the species we examined in this study, the basal margins of the blade were thought to curl downwards (Heckroth, 2004). However, no observations on processes of domatium formation in this species have been carried out. This prompted us to examine the phenomenon in more detail.

6 *Callicarpa saccata* is endemic to Borneo and the ant inhabitation of its domatia was first reported in 1967 (Van Steenis, 1967). *Callicarpa saccata* is the sole foliar domatium-bearing species in the genus *Callicarpa* and its domatia have been described as sac-like, myrmeco-leaf pouches (Janka *et al.*, 2000; Bramley, 2009; Nakashima *et al.*, 2016). These domatia are similar to those found in species such as *Tococa guianensis *of the Melastomataceae, an interesting example of parallel evolution (Van Steenis, 1967). The ants that inhabit *C. saccata* are of the genus *Technomyrmex*, and it was reported that different *Technomyrmex* species that associate with *C. saccata* domatia, distinguished based on colour, show different behaviour in terms of aggressiveness and scale-insect cultivation (Janka *et al.*, 2000). For these ants, domatia appear to function as nurseries: worker ants can be found on blades inside and outside domatia, and cocoons and larvae are found mainly inside these structures (Nakashima *et al.*, 2016), where they are more sheltered. In addition, cup-shaped extrafloral nectaries have been observed inside *C. saccata* domatia (Van Steenis, 1967; Janka *et al.*, 2000). In return for providing shelter and nutrients, colonized trees are believed to suffer less damage from herbivores (Janka *et al.*, 2000).

The purpose of the present investigation was to reveal the anatomy and developmental mechanism of *C. saccata* domatia. In this study, *C. saccata* was compared with the most closely related but domatia-less species *Callicarpa subaequalis*, which was first described in 2009 (Bramley, 2009). This species also has soft, red-brown trichomes on the stems and leaves and develops elliptical leaves with dentate margins, as *C. saccata* has (Bramley, 2009). The use of a closely related reference species represents a strength of this study.

This investigation considered two hypotheses for the developmental processes of domatium formation. The first is that leaf margins curl downwards towards the abaxial surface. This ‘curling blade’ hypothesis is based on observations on *H. physophora* (Leroy *et al.*, 2010) and descriptions of domatia revoluta (Stace, 1965). In this case, a stage of intermediate curling should be observed between flat lamina and enclosed domatium. The second hypothesis is that cell proliferation at the blade/petiole junction causes tissues in this region to warp and grow outwards, creating hollow cavities. In this scenario, the domatium tissues would gradually increase in size without topological change and domatia would therefore develop as structures that are closed at the proximal end. We call this the ‘warping’ hypothesis, and this idea has not been proposed for any species. This hypothesis was based on the fact that the domatia of *C. saccata* and many of the aforementioned domatium-bearing species are located at blade/petiole junctions. This region is an important meristematic site in simple leaves of many angiosperms, supplying cells to both the proximal and distal regions of developing organs (Ichihashi *et al.*, 2011; Tsukaya, 2018). To judge which hypothesis is correct, we examined the developmental processes of *C. saccata* domatia in comparison with the leaves of *C. subaequalis* under cultivation without ants. We also examined glands and inner structures of domatia under the same culture conditions.

## MATERIALS AND METHODS

### Specimen collection, growth conditions and measurements

**Fig. 1.**
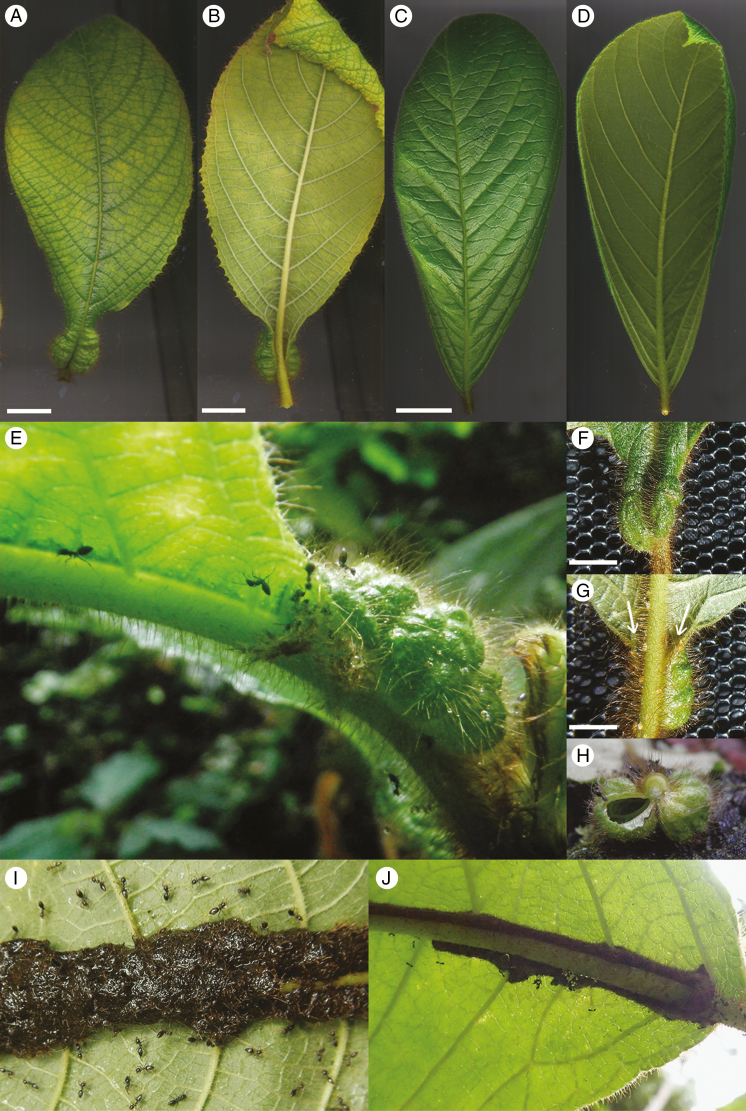
*Callicarpa saccata* and *C. subaequalis* leaves and domatia in their native habitat. Images of mature *C. saccata* (A, B) and *C. subaequalis* (C, D) leaves from the adaxial (A, C) and abaxial (B, D) sides. Scale bars = 2 cm. (E) Lateral view of the proximal region of a *C. saccata* leaf with ants surrounding the domatium. (F, G) Mature domatium from the adaxial side and abaxial side. (H) Transverse view of a domatium, cut open on one side. (I, J) Leaves of *C. saccata* and *C. subaequalis* from the abaxial sides, showing ant-built aisles of organic materials along midveins. Photographs were taken in the native habitat of *C. saccata* (Betung-Kenihun National Park, West Kalimantan, Indonesia, Borneo) by H.T. on 31 December 2011 (E), 6 May 2016 (F, G), 2 January 2011 (H) and 6 May 2016 (I), and the photograph in panel (J) was taken in Kelian Nature Reserve on 9 October 2017.

*Callicarpa saccata* (Fig. 1A, B, E, F, G, H) and *Callicarpa subaequalis* (Fig. 1C, D) are trees that grow close to rivers in the lowland rainforests of Borneo. We first examined *C. saccata* trees in Betung-Kerihun National Park, and collected specimens of the closely related *C. subaequalis* from the nearby Kelian Nature Reserve, both in Kalimantan, Borneo, Indonesia. *Callicarpa saccata* and *C. subaequalis* were collected by H.T. and A.S. in Betung-Kerihun National Park, Indonesia (31 December 2011; permit number 393/SIP/FRP/SM/XII/2011) and Kelian National Reserve, Indonesia (9 October 2017; permit number 338/SIP/FRP/E5/Dit.KI/VII/2017), respectively. Seedlings of both species were cultivated at the University of Tokyo. At the time of measurement of domatia growth, leaf primordia that were too small to have visible domatia were excluded. Domatia were measured on both sides of the midvein, and the mean was used as the value of domatium length.

### Sectioning

In order to observe the growth of domatia, samples were taken of leaf primordia of various sizes and divided into equal halves for sectioning in transverse and longitudinal orientations using the Technovit^®^ 7100 kit (Heraeus Kulzer, Hanau, Germany). For serial sectioning, a 1.6-cm-long leaf primordium was used. The samples were fixed overnight in formaldehyde–acetic acid–alcohol (FAA; 225 mL EtOH, 12.5 mL acetic acid, 32.8 mL formaldehyde and 230 mL H_2_O) and dehydrated in an ethanol concentration gradient (50, 50, 60, 70, 80, 90, 95 and 100 % for 30 min each, and 100 % overnight). The samples were transferred to a 1:1 EtOH and Technovit I solution until they were completely immersed (about 4 h later), and then transferred to 100 % Technovit overnight at −20 °C. Next, the samples were embedded in trays using a 14:1 mixture of Technovit I solution and Hardener II. Once set, a 2:1 mixture of 3040 powder and Technovit Universal Liquid was used to attach the samples to plastic stands. After being removed from the trays, the samples were sectioned using a microtome (Microm HM360, Dreieich, Germany) at thicknesses of 8–12 μm. The sections were stained with 0.1 % (w/v) toluidine blue dissolved in phosphate buffer (pH 7.0). Using a Leica DM4500 B light microscope, images of transverse sections were obtained using a ×10 magnification lens, and images of glands were obtained using a ×100 magnification lens with Leica Immersion Oil (Leica Microsystems™, Wetzlar, Germany).

### Micro-CT scanning

To further observe domatium development, micro-X-ray CT scanning was carried out at The University Museum, The University of Tokyo, using a ScanXmate B100TSS110 scanner (Comscan Tecno, Yokohama, Japan). The sample was a 2-cm-long *C. saccata* leaf primordium, which was fixed overnight in FAA (as above) before being stained with 1 % (w/v) iodine to improve visualization. The scan parameters were a tube voltage of 100 kV and a tube current of 29 µA. The size of the detector was 1024 × 1012 pixels and resolution was 4.437 μm.

### Chemical composition of secretions

To assess the chemical composition of glandular secretions, domatia were first cut in half, after which the secretions within could be seen with the naked eye. The secretion (3 µL) was collected directly from the domatium and diluted to 1:10^14^ in extraction solution (0.1 % formic acid in 80 % MeOH). The diluted secretion (1 µL) was subjected to analysis by liquid chromatography–triple quadrupole mass spectrometry (LC-QqQ-MS). A total of 516 metabolites, determined as described previously (Sawada *et al.*, 2009) was assayed by LC-QqQ-MS-based widely targeted metabolomics.

### Scanning electron microscopy and gland density analysis

To observe the glands of *C. saccata* and *C. subaequalis*, 1-cm^2^ sections were cut from one mature blade of three *C. saccata* and three *C. subaequalis* individuals for surface observations. Three domatia were split in half, and the glands in one half were enumerated and measured. Each half was divided into six sections for observation. The samples were fixed overnight in FAA (as above) and dehydrated in a graded EtOH series (50, 50, 60, 70, 80, 90, 95 and 99.5 %) for 30 min per step, followed by 100 % EtOH overnight. The samples were transferred to a 1:1 EtOH and isoamyl acetate solution for 30 min, and washed twice with 100 % isoamyl acetate for 15 min each. The samples were critical-point-dried using a JCPD-5 critical-point dryer (JEOL Datum, Tokyo, Japan), and subsequently sputter-coated for 90 s at 20 mV using a JEC-300FC auto-fine coater (JEOL Datum). Finally, the samples were observed under a JSM-6510LV scanning electron microscope (JEOL Datum). The sizes of round, star-shaped and cupulate glands were measured, and their densities were calculated across the adaxial and abaxial surfaces of *C. saccata* and *C. subaequalis*. In the case of *C. saccata*, the gland density inside and outside of the domatia was assessed. Density was calculated as the number of glands on 1 cm^2^ of tissue and in relation to the number of pavement cells. In each category, three mature leaves were observed. Average gland diameter was determined using ImageJ (National Institutes of Health and the Laboratory for Optical and Computational Instrumentation, WI, USA).

## RESULTS

### Growth

**Fig. 2.**
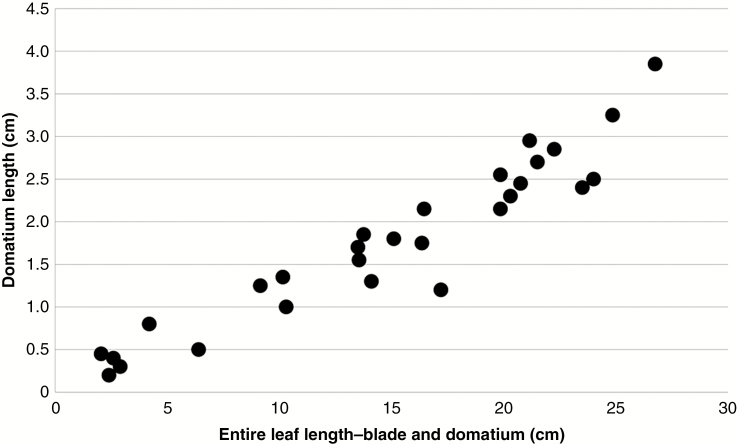
Scatterplot of the growth of *C. saccata* blades and domatia. The *x* axis shows the total length of blades and domatia (cm), measured from the distal tip of each leaf to the proximal end of each domatium. The *y* axis shows domatium length (cm). Twenty-eight leaves were measured.

In its native habitat, *C. saccata* associates closely with ants that can live inside the hollow cavities of domatia (Fig. 1H). The ants use organic materials to build habitable living spaces along midveins (Fig. 1I), as reported by Janka *et al.* 2000. In a rare case, this behaviour was observed on the leaves of one *C. subaequalis* individual (Fig. 1J). To observe domatium growth in *C. saccata*, we firstly cultivated several seedlings under laboratory conditions (23 °C, continuous illumination with fluorescent lamps at about 60 μmol m^−2^ s^−1^) and compared them with *C. subaequalis* that were under cultivation. After seed germination, we found that the first two nodes of all *C. saccata* individuals developed domatia-less leaves (*n* = 7), whereas all leaves on subsequently emerging nodes developed domatia. Domatium formation was seen under cultivation without symbiosis with ants, clearly indicating that domatium morphogenesis is independent of ants. Domatia grew proportionately to leaves, reaching lengths of up to 3.5 cm (Fig. 2). On average, domatia accounted for 10.8 % of total leaf length. On the contrary, no domatium formed on the 87 *C. subaequalis* leaves observed (*n* = 5 individuals).

**Fig. 3.**
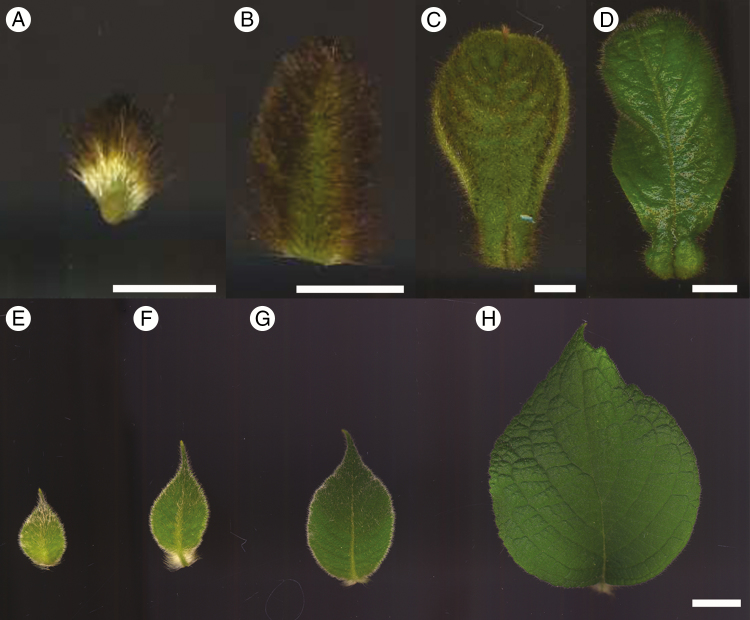
Development of *C. saccata* and *C. subaequalis* leaves under cultivation. Developing leaves of *C. saccata* (A–D) and *C. subaequalis* (E–H) from the adaxial side. Scale bars = 0.5 cm.

Figure 3 shows developing leaves of *C. saccata* (A–D) and *C. subaequalis* (E–H); the domatia of *C. saccata* are visible in the proximal regions of the blades (Fig. 3C, D). Each domatium comprises a cavity on either side of the midvein (Fig. 1H). Domatia always protrude outwards on the adaxial side, with openings on the abaxial sides through which insects are able to enter, at the distal points of the structures (Fig. 1G). As small primordia are densely covered in trichomes, small developing domatia cannot be seen with the naked eye; therefore, leaves were sectioned to enable observation of domatia at an early stage of development.

### Domatium development

**Fig. 4.**
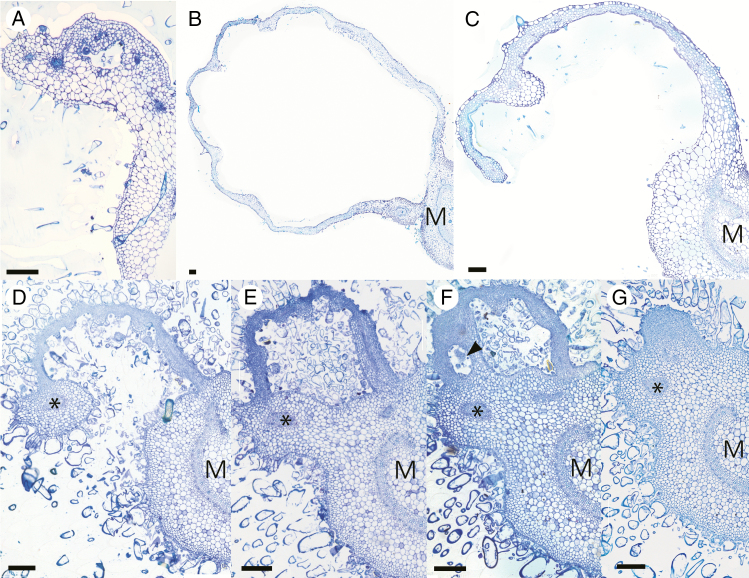
Comparison of developing *C. saccata* domatia and proximal blade tissue of *C. subaequalis*. Cross-sectional images of a young (A) and a more mature (B) *C. saccata* domatium. The young primordium was 1.2 cm in length and the domatium of the more mature leaf was 1.1 cm. Cavities increase in size as leaves age. (C) Corresponding proximal region of a *C. subaequalis* leaf. (D–G) Serial cross-sectional images of a single *C. saccata* domatium. The domatium is open at the distal end (D) but closed at the proximal end (G). A small cavity, indicated by an arrowhead, is part of the larger open space (F). M, midvein; *, lateral vein. Images are composite. Scale bars = 200 μm.

We then attempted to determine how cavities are formed. Figure 4 shows the small domatium cavity of a young leaf primordium (Fig. 4A, D–G) and a structural comparison of a larger *C. saccata* domatium (Fig. 4B) with the corresponding basal region of a *C. subaequalis* leaf blade (Fig. 4C). Serial sectioning of primordia (Fig. 4D–G) demonstrated that domatia are open at the distal ends (Fig. 4D), allowing ants to enter. The cavities gradually increase in size without topological change during the course of domatium development (Fig. 4A, B) and no intermediate form indicating curling was observed. The domatia are sandwiched between the midvein and lateral veins of blades (Fig. 4D–G, lateral veins indicated by asterisks) and the domatia margins end with the lateral veins, as shown in Fig. 4. The sections also showed that cell proliferation, indicated by the presence of small, cytoplasm-dense cells, is active between the midvein and the lateral veins, at the distal end of domatia, localized to the base of the leaf lamina. This local cell division seems to push the leaf laminas upwards over the veins. Early cavities made by such cell proliferation were observed in primordia as small as 0.6 cm in length, but before this stage domatium formation was not observed. In *C. subaequalis*, the leaf blades lay relatively flat (Fig. 4C).

**Fig. 5.**
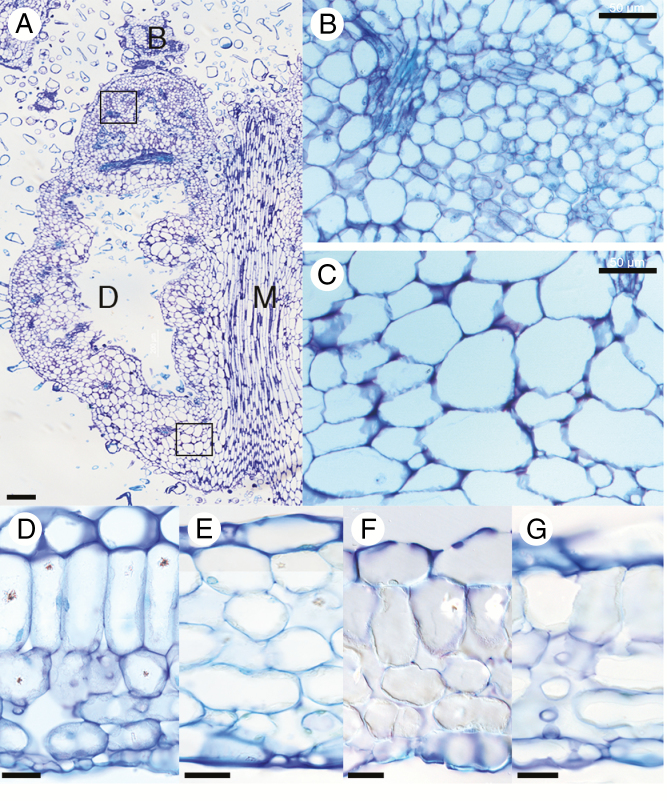
Histology of domatia. (A) Paradermal section of a still-growing *C. saccata* domatium (length 3 mm). ‘B’, ‘D’ and ‘M’ refer to the blade, domatium and midvein. (B, C) Magnified views of the distal region and proximal region. The locations of cells shown in (B) and (C) in the domatium are indicated by the black boxes in (A). (D–G). Sections of a *C. saccata* blade (D) and domatium (E), and sections of a *C. subaequalis* blade (F, G). The *C. saccata* blade section was taken from the central region of the lamina, and the *C. subaequalis* sections were taken from the proximal (F) and distal (G) regions. Note that the blades (D, F, G) show ordinary cell layers, whereas the domatium (E) does not. Scale bars = 50 μm (A), 20 μm (B, C) and 50 μm (D–G).

To confirm the localized cell proliferation activity at the distal end of domatia, paradermal sections of young primordia were also made. A mature domatium was divided along the midvein and one half was sectioned (Fig. 5A). The cells of the distal region (Fig. 5B) are smaller than those of the proximal region (Fig. 5C), showing that cell division is biased in the proximal direction and tissue is pushed outwards over the petioles, with the site of cell proliferation maintained at the blade/domatium boundary.

**Fig. 6.**
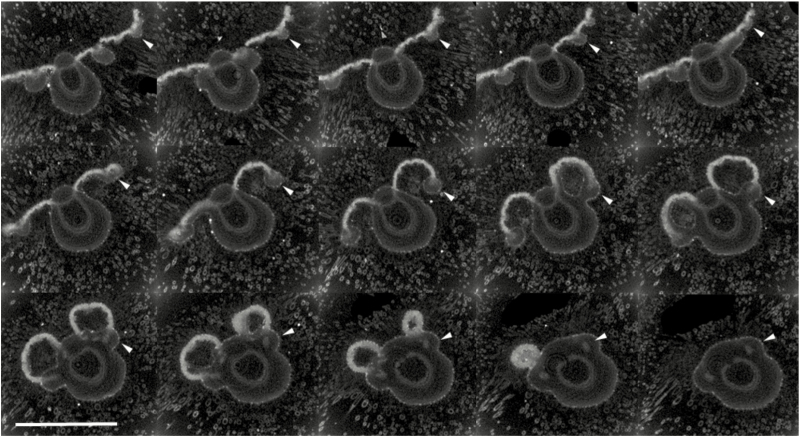
Micro-CT images of a 2-cm-long *C. saccata* domatium from the distal (upper left) to the proximal end (bottom right). In the more distal regions the blade tissue is relatively flat. Towards the proximal end the blade tissue is curved, as cell proliferation in the proximal zone has warped its shape. Arrowheads indicate lateral veins, approaching the midvein in the proximal region. In the final two images, the domatium can be seen to decrease in size towards the petiole and to be closed at the proximal end. Scale bar = 1 mm.

In order to confirm the above observation, primordia were subjected to X-ray micro-CT to further evaluate the mechanism of domatium development. Selected images show the structure of the leaf from the distal to the proximal areas (Fig. 6). The distal regions of the blade are flat. Towards the proximal end, the blade appears to be curved, as cell proliferation in the proximal zone warps its structure. Finally, two enclosed cavities are seen (Fig. 6), decreasing in size towards the petiole and eventually disappearing. Importantly, the margins of the cavities are always contiguous with lateral veins, as indicated by asterisks in Fig. 4D–G and arrowheads in Fig. 6. Through X-ray micro-CT scanning, we observed that domatium formation is not seen at the earliest stages of leaf development (Supplementary Data Fig. S1), indicating that excess cell proliferation in a biased direction starts after the establishment of primary organogenesis for the leaf lamina and petiole. Through sectioning and CT scanning, we revealed that the growth of domatia was due to cell proliferation at the blade/petiole junction. Then, we analysed the tissue structures of blades and domatia.

### Structure

Observations with the naked eye showed that the domatia retained adaxial features (dark coloration, waxy surface and dense red-brown trichome distribution), and the insides of domatia resemble the abaxial sides of leaf blades (light coloration, sparser trichomes). To know whether such dorsoventral differentiation also occurs at the tissue level, we examined the structure of *C. saccata* domatia in comparison with the blade tissues of the same species and those of *C. subaequalis.* We found that the blades of *C. saccata* and *C. subaequalis* comprise both palisade and spongy layers (Fig. 5D, F, G). In comparison, although the epidermis and mesophyll layers are clearly present in domatia of *C. saccata*, we found that the mesophyll of domatia does not contain elongated palisade tissue (Fig. 5E).

### Glands

Janka *et al.* (2000) reported the presence of cupulate glands on the inner surfaces of the domatia of *C. saccata* in the wild and stated that these glands produce sugars to nourish ants. We found aqueous droplets on the inner surfaces of domatia also from a cultivated *C. saccata* individual grown in the absence of ants, indicating that droplet secretion is ant-independent. We further analysed the contents of the droplets using LC-QqQ-MS, revealing that these secretions were rich in sucrose (Table 1). As no glands have been reported previously in *C. subaequalis*, we compared the gland types present in the two *Callicarpa* species using scanning electron microscopy.

**Table 1.** Chemical composition of *C. saccata* glandular secretion

Name	s/n
Melibiose/turanose/isomaltose/gentiobiose/melibiose/palatinose	82
Sucrose	47
l-Carnitine	18
l-Arginine/*N*α-acetyl-l-ornithine/l-citrulline	15
Trigonelline	15
Guanine	12
l-Tyrosine	12
l-Lysine	11
Adenine	10

s/n, signal-to-noise ratio of *C. saccata* secretion, measured by LC-QqQ-MS.

**Fig. 7.**
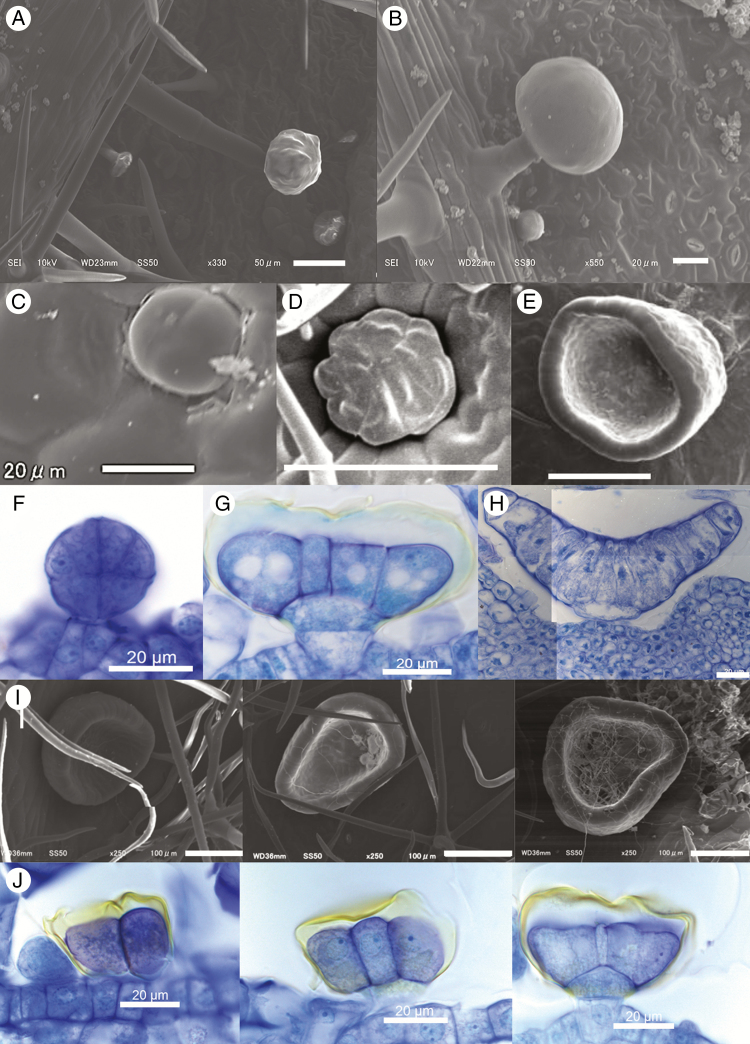
(A–F) Scanning electron microscope and light microscope (G–J) images of glands observed in *C. saccata* and *C. subaequalis* blades. Peltate (A), capitate (B), round (C), star-shaped (D, H) and cupulate glands (E, F, I) are shown. Images are of the abaxial surface of *C. subaequalis* blades (A, B, J), the abaxial surface of *C. saccata* blades (C, D, G, H) and the inner surface of *C. saccata* domatia (E, F, I). Scale bars = 50 μm (A), 20 μm (B, C, F–H, J) and 100 μm (D, E, I).

We recognized three gland types in *C. saccata* and *C. subaequalis.* Small, round glands (Fig. 7C) were 330–695 μm^2^ in diameter (Table 2) and composed of eight cells (Fig. 7F). We observed these small, round glands on the adaxial and abaxial surfaces of the blades of *C. saccata* and *C. subaequalis* and on the inner and outer surfaces of *C. saccata* domatia. On the abaxial surface of blades, they were found at densities of 175 per 1 cm^2^ on *C. saccata* and 532 per 1 cm^2^ on *C. subaequalis*; the density was thus greater on domatia-less *C. subaequalis.* On the adaxial surfaces of leaves, round glands were found at densities of 637 per 1 cm^2^ in *C. saccata* and 414 per 1 cm^2^ in *C. subaequalis.* On the outer and inner surfaces of *C. saccata* domatia they were found at densities of 80 and 52 per 1 cm^2^, respectively (Table 2).

**Table 2.** Gland size and density in *C. saccata* and *C. subaequalis*

Species			*C. saccata*		*C. subaequalis*
Position			Blade	Domatium	Blade
Round glands	Size (µm^2^)	Abaxial	390 ± 15.5	547.7 ± 35.1	546.2 ± 117
		Adaxial	485 ± 21.5	695.1 ± 24.7	338.3 ± 10.7
	Density (number in 1 cm^2^)	Abaxial	175	52	532
		Adaxial	637	80	414
	Number in domatium		N/A	705	N/A
Star-shaped glands	Size (µm^2^)	Abaxial	3707.5 ± 182.3	3764.5 ± 228.5	3349.4 ± 71.2
	Density (number in 1 cm^2^)	Abaxial	116	44	949
	Number in domatium		N/A	78	N/A
Cupulate glands	Size (µm^2^)	Inner domatium	N/A	30 876.9 ± 3527	N/A
	Density (number in 1 cm^2^)	Inner domatium	<1 Rare	12	<1 Rare
	Number in domatium		N/A	176	N/A

Size data are mean ± standard deviation.

N/A, gland was not present or not counted in a particular sample type.

Secondly, star-shaped glands in *C. saccata* were previously reported by Nakashima *et al.* (2016). We found these star-shaped glands on the abaxial surfaces of the leaves of both *C. saccata* and *C. subaequalis* (Fig. 7D). Additionally, they were observed on the inner surface of *C. saccata* domatia. Star-shaped glands averaged 3349 μm^2^ in C. *subaequalis*, 3707 μm^2^ on the blades of C. saccata and 3764 μm^2^ on the domatia of C. *saccata* (Table 2). They comprised a single basal epidermal cell, a single stalk cell and an eight-cell upper secretory structure. The secretory structure supported a storage cavity surrounded by a cuticle (Fig. 7G). On the abaxial surface of blades, they were found at a density of 116 per 1 cm^2^ in *C. saccata* and 949 per 1 cm^2^ in *C. subaequalis.* On the inner surface of *C. saccata* domatia, they were present at a density of 44 per 1 cm^2^.

Lastly, large, cupulate glands (Fig. 7E) were previously reported to be present inside *C. saccata* domatia (Janka *et al.*, 2000; Nakashima *et al.*, 2016). Janka *et al.* (2000) observed these glands through sectioning of *C. saccata* domatia and proposed that these glands produce sugars to attract ants, particularly on younger branches. In this study, we found these glands on the inner surface of *C. saccata* domatia, very rarely on the outer surface and not on the blades. Cupulate glands averaged 30 877 μm^2^ in area and were each composed of a basal epidermal cell, a stalk cell and a complex upper structure of elongated cells (Fig. 7H). On the inner surface of *C. saccata* domatia, they were found at a density of 12 per 1 cm^2^. Cupulate glands were absent in domatia-less *C. subaequalis.* Cupulate glands of various sizes were observed on the inner domatium surface (Fig. 7I), and developing star-shaped glands were observed in sections of a *C. subaequalis* blade (Fig. 7J).

## DISCUSSION

In the present study, we investigated the developmental processes of *C. saccata* myrmeco-domatia in comparison with the related domatia-less reference species *C. subaequalis.* We determined that excess cell proliferation at the blade/petiole junction produces the tissues of domatia. This over-proliferation apparently causes a warping of flat laminas, and tissues expand outwards over petioles. This is further supported by a saucer-like, incomplete form of *C. saccata* domatia, which has been observed at the early nodes of young seedlings (Supplementary Data Fig. S2). The timing of domatium appearance may be important for maintaining the ant–plant relationship (Leroy *et al.*, 2010). Except for leaves at nodes 1 and 2, all *C. saccata* leaves developed a domatium.

**Fig. 8.**
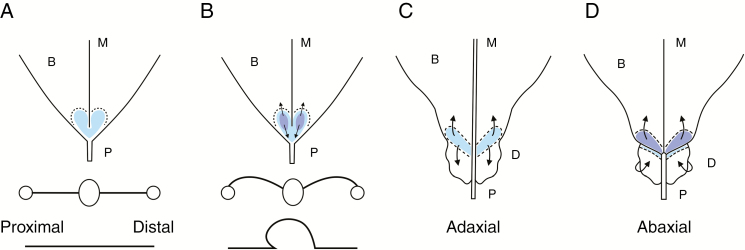
Genesis of domatia. Schematics showing the proximal region of a *C. saccata* leaf from the adaxial (A–C) and abaxial (D) sides. Transverse and longitudinal schematics are shown below (A) and (B), with large circles representing midveins and small circles representing lateral veins. The site of the blade/petiole junction is shown in pale blue, as viewed from the adaxial side (A–C). This proliferative site on the adaxial side, viewed from the abaxial side, is shown in dark blue in panel (D). This site of cell proliferation in simple leaves overlaps with the site of domatium formation. The lamina is initially flat (A) before tissues begin to grow outwards (B). Arrows indicate the direction of cell supply. Over-proliferation in this region, in the biased manner indicated by patterns of cell division, results in domatium growth across the petiole. B, blade; M, midvein; D, distal; P, proximal.

This finding contradicts the previous idea of domatium formation in *C. saccata* as a ‘curling under’ of leaf margins (Heckroth, 2004) but supports the warping hypothesis. Both ‘curling’and ‘warping’ likely rely on excess cell division; warping is hypothesized to be due to cell division in a laterally polarized direction, while warping is due to longitudinal polarization. We summarize our understanding of domatium formation by the schematics in Fig. 8. In panels (A) and (B), transverse schematics are shown in which large circles represent midveins and small circles indicate the positions of lateral veins. A longitudinal view from the proximal side to the distal side of the blade/petiole junction is shown below panels (A) and (B). The blade tissue extends upwards (Fig. 8B) and is eventually pushed outwards over the petiole (Fig. 8C), creating hollow structures between the midveins and the leaf margins. It is likely that the domatia of species including *M. guianensis* and *T. guianensis* develop in the same way. A comparison between *M. guianensis*, *T. guianensis* and *C. saccata* will be the focus of a future investigation. Similar polar-biased cell proliferation in leaf primordia can result in the formation of cup-shaped, pouch-like or tube-like structures of leaves also in remotely related taxa, such as *Cinnamomum camphora* and *Sarracenia purpurea* (Nishida *et al.*, 2006; Fukushima *et al*., 2015). Thus, organogenesis of tubular or dome-like shapes mediated by cell proliferation might be a common strategy in angiosperm leaves.

We also found that the mesophylls of *C. saccata* domatia did not contain elongated palisade cells, which has been reported in *H. physophora* of the Chrysobalanaceae and *T. guianensis* of the Melastomataceae. The domatia of *M. guianensis* show limited palisade differentiation (Leroy *et al.*, 2008, 2010). These findings suggest that the primary role of domatia is not in photosynthesis, which requires a well-differentiated palisade layer, but in support of the ant–plant relationship. The development of similar sac-like foliar domatia in these distantly related families also indicates that these structures represent an interesting case of parallel evolution (Van Steenis, 1967). The determination of whether abaxial–adaxial identities are established in domatia, as they are in leaf blades, would be of interest. If so, a future line of investigation would be to determine how palisade tissue differentiation is suppressed in domatia.

In the present study, we also examined the variety and density of glands in *C. saccata* and its close relative, *C. subaequalis*. We found capitate and peltate glandular trichomes on the blades of both *C. saccata* and *C. subaequalis.* Cupulate glands were previously observed on the inner surfaces of *C. saccata* domatia (Van Steenis, 1967; Janka *et al.*, 2000; Nakashima *et al.*, 2016), and we confirmed these glands on the outer surfaces of *C. saccata* domatia, while these glands were absent on the leaf blades. Janka *et al.* (2000) proposed that cupulate glands produce sugars that are attractive to ants. We also detected sucrose in droplets from the domatia of *C. saccata* under our laboratory conditions (Table 1), indicating that sucrose secretion is ant-independent. The interesting distribution of cupulate glands in *C. saccata,* combined with the lack of cupulate glands in *C. subaequalis*, suggests a unique role for this gland type.

Previously, several reports described glandular trichomes in *C. saccata* but gland density was not previously reported. Here we revealed the lower densities of round and star-shaped glands on the abaxial surfaces of *C. saccata* leaves, suggesting that the presence of a domatium reduces the need for these glands.

Species in the mint family (Lamiaceae) secrete monoterpene-containing aromatic oils from capitate and peltate glandular trichomes such as those seen in *C. saccata* and *C. subaequalis* (Fig. 7A, B). Such glandular trichomes have been reported to release herbivore-deterring essential oils in various Lamiaceae species (Turner *et al.*, 2000; Haratym and Weryszko-Chmielewska, 2017; Schmiderer *et al.*, 2007; Giuliani *et al*., 2017). Therefore, the lower density of such glands in *C. saccata*, if they do indeed secrete terpene, may have evolved to create a more habitable living space for ants. The negative effects of reduced secretion of insect repellent may be counteracted by the protection provided by the resident ants.

Unexpectedly, despite the absence of domatia in *C. subaequalis*, we found that this species is at least in part a myrmecophyte, although ant-built structures were only observed in a single population/tree (Fig. 1J). If ants are attracted by sucrose, as in *C. saccata*, the identity of the sucrose-secreting gland awaits confirmation. Alternatively, as soft, red-brown trichomes are shared by these two species (Bramley, 2009), the presence of these hairs might be a key character in attracting ants to build their nests. Janka *et al.* (2000) wrote that presence of hairs around the openings of *C. saccata* domatia may limit the size of ants that are able to enter. To determine this, large-scaled ecological studies in their native habitats are needed in the future.

*Callicarpa saccata* is an interesting species of the genus *Callicarpa*, as it bears pouch-like foliar domatia that form independently of colonizing ants. *Callicarpa saccata*-like domatium morphology has been documented in numerous species (Janka *et al.*, 2000; Izzo and Vasconcelos, 2002; Leroy *et al.*, 2008, 2010). Here we revealed the developmental processes of *C. saccata* domatia and found that the blade/petiole junctions of leaves are sites of excess cell proliferation. This proliferation pushes the blade upwards in the adaxial direction and outward in the distal direction, resulting in the formation of two hollow cavities on either side of the midvein, within which ants are able to live. Excess cell division in the basal region warps the shape of the developing lamina. Finally, we observed three types of gland in *C. saccata* and compared these glands with those of *C. subaequalis.* Research on the gene expression network in developing domatia would allow us to understand the molecular mechanisms that alter leaf morphology and support mutualistic relationships with ants. Knowledge of the genetic control of domatium formation in *C. saccata* would enhance our understanding of the molecular mechanisms of domatium development.

## SUPPLEMENTARY DATA

Supplementary data are available online at https://academic.oup.com/aob and consist of the following.

Figure S1: young *C. saccata* leaf primordia, observed by CT scanning. No domatia are observed at this stage. Midveins are indicated by ‘M’. Scale bar = 0.5 mm.

Figure S2: young *C. saccata* leaf from the adaxial and abaxial sides. This leaf was taken from the third node of a *C. saccata* seedling. The saucer shape of the domatium is clear from the abaxial side. Scale bar = 1 cm.

mcz193_suppl_Supplementary_Figure_S1

mcz193_suppl_Supplementary_Figure_S2

mcz193_suppl_Supplementary_Legends
