# Supplementary figures and images for: Morphological characterization of domatium development in Callicarpa saccata
Source: Ann Bot. 2019 Nov 26;125(3):521–32. doi: 10.1093/aob/mcz193 (PMC7061170; doi:10.1093/aob/mcz193)

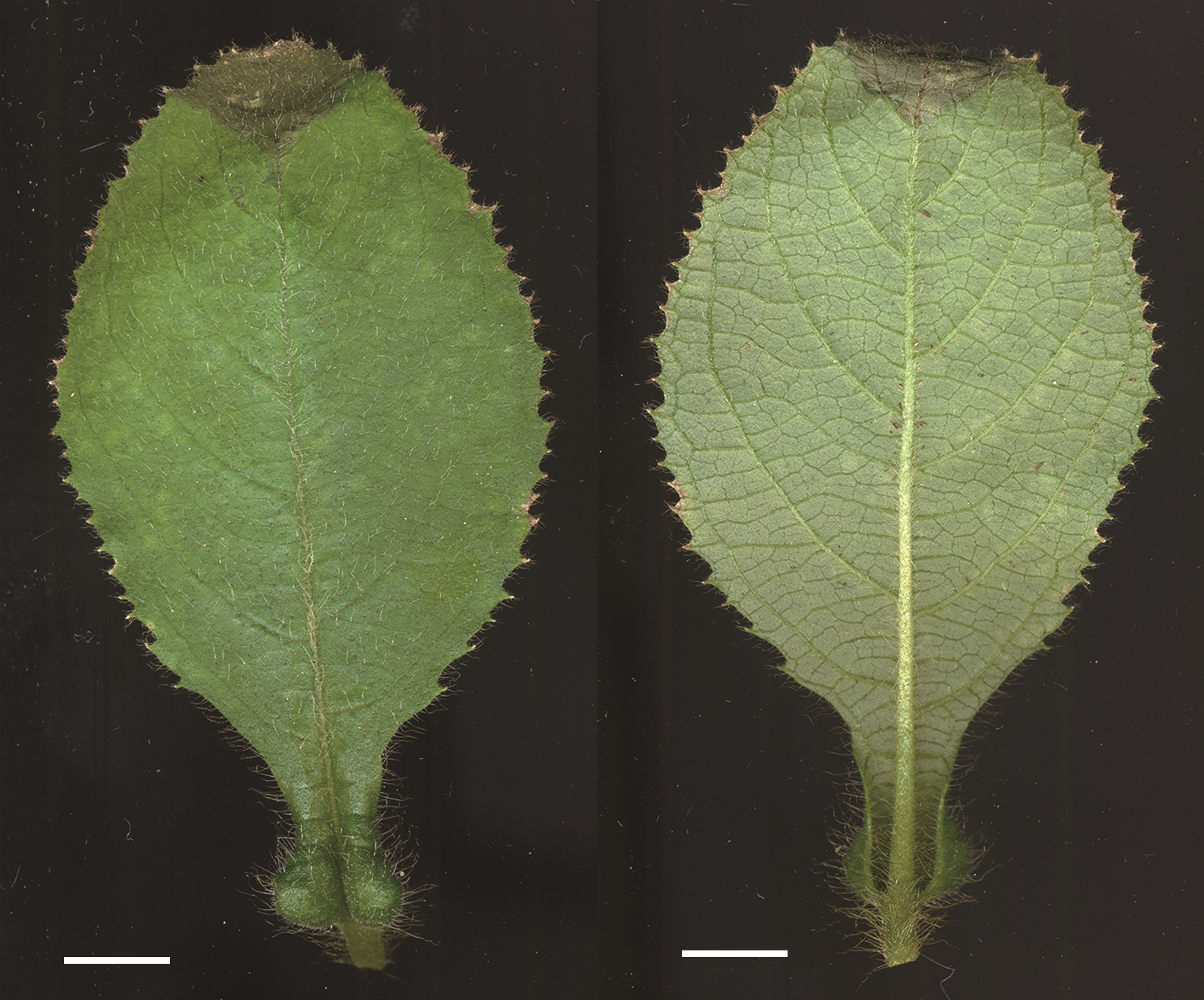

Supplement: mcz193_suppl_Supplementary_Figure_S1 [file mcz193_suppl_supplementary_figure_s1.png]

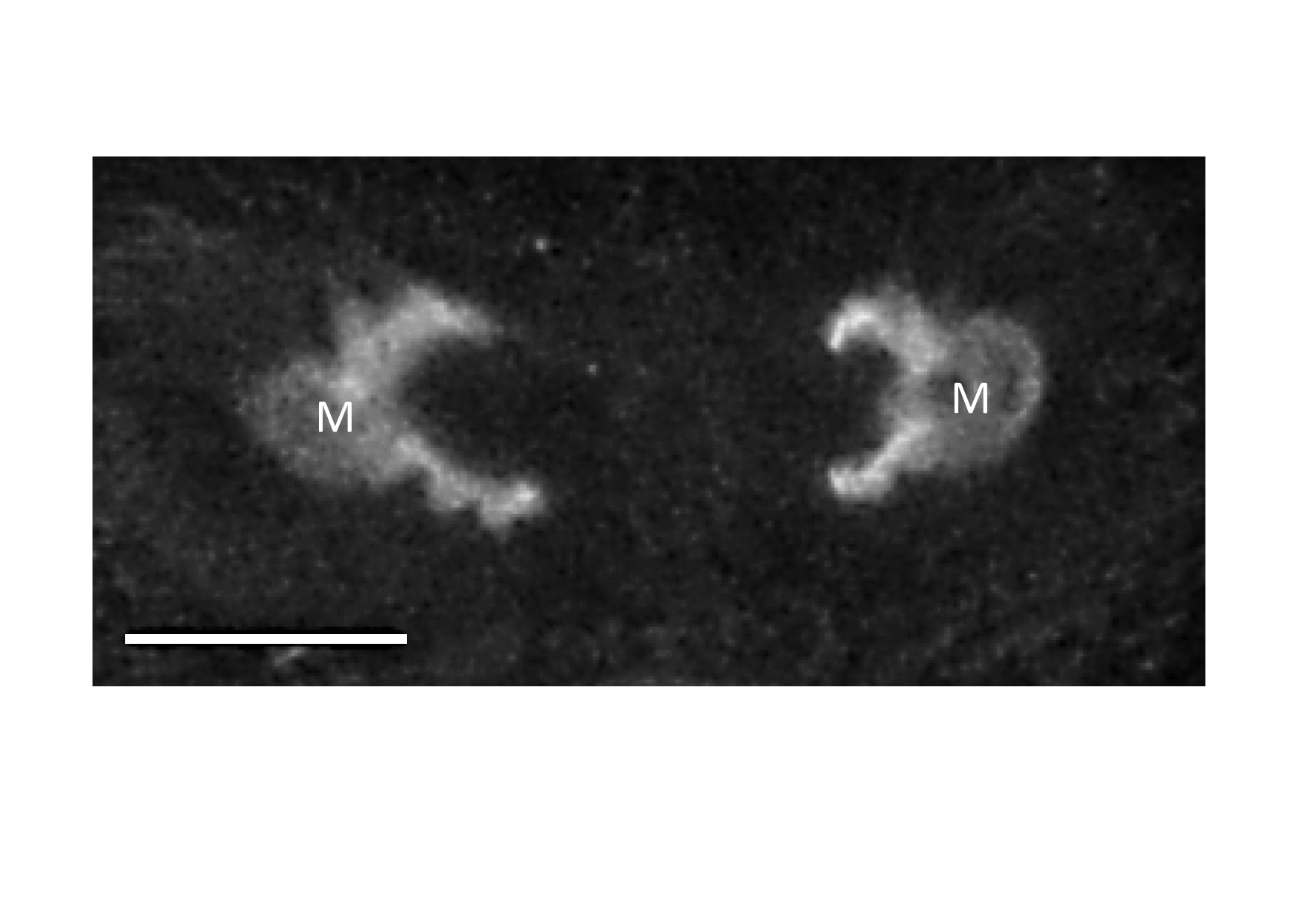

Supplement: mcz193_suppl_Supplementary_Figure_S2 [file mcz193_suppl_supplementary_figure_s2.png]
