# Supplementary material for: Morphological characterization of domatium development in Callicarpa saccata
Source: Ann Bot. 2019 Nov 26;125(3):521–32. doi: 10.1093/aob/mcz193 (PMC7061170; doi:10.1093/aob/mcz193)
Supplement: mcz193_suppl_Supplementary_Legends [file mcz193_suppl_supplementary_legends.docx]

Supplementary data captions

Fig. S1 shows young *C. saccata* leaf primordia, observed through CT scanning (transverse view). No domatia are observed at this stage. Midveins are indicated by ‘M.’ Scale bar = 0.5 mm.

Fig. S2 shows a young *C. saccata* leaf from the adaxial (A) and abaxial (B) side. This leaf was taken from the 3^rd^ node of a *C. saccata* seedling. The saucer-shape of the domatium is clear from the abaxial side. Scale bar = 1 cm.
